# The Demand for Reconstruction

**Published:** 1919-11-22

**Authors:** 


### SOME REMARKS ON MEDICAL EDUCATION.*

### The Demand for Reconstruction.

Sir George Makins' thoughtful address is welcome and opportune. There is an urgent demand for well-considered reconstruction, and this is essentially the time to secure the necessary support. Unfortunately, the desire for progress is too often expressed in spasmodic and isolated efforts by individuals and institutions, whose motives are not invariably unmixed. It is therefore with real interest that we turn to the unbiassed observations of an acknowledged authority.

### Two Main Problems.

There are two main problems: the education of the undergraduate, who is gradually being forced to acquire the attributes of a mere encyclopædia, and the education of the graduate, for whom there is no really systematic provision at the present time. With regard to the former, Sir George Makins insists on our aiming at the production of an efficient practitioner, rather than a type of individual crammed with disconnected tags of knowledge. There is much to be said for the ancient course of apprenticeship. The progress of knowledge, combined with the frequency of examinations, especially that curse of modern education the competitive examination, is tending to inflict on the student a task well-nigh impossible, and to destroy any possibility of his acquiring a well-proportiond view of the general principles of medicine. It is necessary to distinguish clearly between the practitioner, however intelligent and logical his reasoning, and the student of pure science. The further we advance, the wider the gap, and the more important the function of the teacher, who, unhandicapped by private practice and routine duties, can keep in touch with the laboratories, competently criticise the value of new discoveries, and impress the importance of their application in the wards. "In the education of the doctor, medicine itself must be allowed to take a more prominent part as an exercise of scientific thought and method, and the attempt to develop a scientific turn of mind by administering smatterings of the auxiliary sciences should be abandoned."

### A Re-grouping of Subjects.

A regrouping of subjects is suggested as a remedy for some of the present evils, together with a general simplification:

"Elementary biology as a separate course to be excluded from the curriculum, and the congeries of subjects dealt with in the existing syllabus relegated to their respective positions at the commencement of the courses of anatomy, physiology, and pathology. The first year of study to be devoted to anatomy, chemistry, and physics. The second year to be devoted mainly to physiology, together with classes in anatomy so arranged that the subjects dealt with run concurrently with those to which they relate in the course of physiology. The third year to be devoted to the study of pathology and pharmacology, including such elements of the course of materia medica as may be retained. During the summer session of this year elementary instruction in clinical methods. The fourth and fifth years to be entirely devoted to professional subjects as at present. An examination in anatomy and physiology at the end of the second year. No examination in chemistry and physics to be held by the licensing bodies, but the whole responsibility for sufficient instruction in these subjects to be thrown upon the respective teachers in the schools, who would satisfy themselves by means of class examinations. An examination in pathology and pharmacology at the end of the third year. The final examination in professional subjects to be held as at present after the termination of the fifth year."

### The Degrees of Importance.

Naturally, we may disagree with details of this scheme according to the degree of importance each of us attaches to the various subjects. Few will object to the curtailing of time devoted to the minor points of anatomy: it may be a source for regret that the dissecting room has ceased to be the centre of life in a medical school; but this is rendered inevitable by the need for economy in time and energy, and also by the scarcity of material. Yet it would seem dangerous to limit the study of such vital subjects as physiology and *general* biology. No man equipped with a knowledge of their principles can make many serious mistakes in medical practice; it is rather the man who relies on a memory of individual cases who will find himself at a complete loss when confronted with the unusual.

However, none can disagree with the main objective. The essentials are simplification, and, above all, continuity and co-ordination. Re-adjustment of the various departments and collaboration will be necessary to obtain a full advantage.

The existing system of *clinical* instruction is not adversely criticised, but a plea is entered for the utmost simplification in methods of diagnosis and the need for exactitude of observation. The habitual resort to special methods is deprecated as likely to destroy the art of the practitioner.

### Graduate Instruction.

Turning to the all-important question of graduate instruction, we are impressed with the need for the establishment of a central institution which shall provide for the further training of specialists, teachers, and research workers, affording scope for the systematic acquisition of recently established knowledge and for scientific investigation; and

* An abstract of an address delivered by Sir George Makins before the Manchester Medical Society on October 29, 1919.

**Some Remarks on Medical Education**—(*continued*).

shall also provide for the graduate from the provinces, from the dominions and from abroad, the practitioner who wishes to refresh, modernise and augment his store. Praiseworthy attempts have already been made to supply this obvious demand; but in no cases have the resources of any institution been large enough to meet the future needs. Courses of work suitable for the undergraduate are comparatively useless to the qualified man; whereas the former needs instruction more or less limited and dogmatic, the latter requires an acquaintance with the methods and results of recent research, a knowledge of special literature, and an insight into current controversy. Schools and teachers adapting themselves to the purpose of primary instruction in medicine are ill-suited to the requirements of the graduate student. The question demands solution by the establishment of a system which can co-ordinate investigation, and by the formation of secondary schools of medicine, one of which might form a centre for the whole country.

### Staff and Equipment.

As regards the staff and equipment, the first essential for a graduate school is the possession of a large and well-appointed hospital entirely devoted to the purpose, with sufficiently well-equipped and extensive laboratories attached. The staff should be of the professorial type, not, however, composed entirely of permanent whole-time professors. In the case of clinical work, officers from the various existing schools might be seconded for a period of five years, thereby regularly infusing new blood and establishing co-ordination. By this means the undergraduate schools would also benefit, from the additional experience acquired by their teachers, and the standard of education would be generally raised. Sir James Mackenzie argues that the general practitioner is essentially the man to investigate the beginnings of disease: in the secondary school the accomplished general practitioner might find a proper place. The hospital would furnish full opportunities for observation and research and facilities for practical work and technical development of the highest class. Graduates would take the place of the junior officers in an ordinary hospital, acting as assistants to the professors.

### Associations Already at Work.

Associations which already have the work of co-ordination in hand are the proper agency through which the entire scheme could be completed.

The whole wealth of material possessed by the hospitals, infirmaries, laboratories, and museums of the Metropolis would acquire a bond of union. Opportunities and material at present wasted from the educational standpoint would be utilised to the general advantage. The graduate in his search for knowledge would no longer be compelled to waste the greater part of his time and energy in seeking subjects and expert advice from one corner to another, for his school would provide a vast field of action, and bring him in contact with every source of inspiration.

The ideal so ably outlined by Sir George Makins cannot fail to stimulate: the need is obvious. Two essentials are required for its realisation—financial support and the abolition of individual jealousies. The latter may seem almost too great an obstacle to face—but the war should have taught many lessons, even to the medical profession.

